# Hip arthroscopic management for treating a rhythmic gymnast with a large bone cyst at the femoral head in the setting of hip dysplasia—a case report

**DOI:** 10.1093/jhps/hnac021

**Published:** 2022-04-25

**Authors:** Akira Fujiike, Yoichi Murata, Akihisa Hatakeyama, Shinichiro Takada, Akinori Sakai, Soshi Uchida

**Affiliations:** Department of Orthopaedic Surgery, Wakamatsu Hospital of University of Occupational and Environmental Health, Kitakyushu, Japan; Department of Orthopaedic Surgery, Wakamatsu Hospital of University of Occupational and Environmental Health, Kitakyushu, Japan; Department of Orthopaedic Surgery, Wakamatsu Hospital of University of Occupational and Environmental Health, Kitakyushu, Japan; Department of Orthopaedic Surgery, Wakamatsu Hospital of University of Occupational and Environmental Health, Kitakyushu, Japan; Department of Orthopaedic Surgery, University of Occupational and Environmental Health, Kitakyushu, Japan; Department of Orthopaedic Surgery, Wakamatsu Hospital of University of Occupational and Environmental Health, Kitakyushu, Japan

## Abstract

Recent literature lacks a clear understanding of how to manage bone cysts associated with hip dysplasia. This article aimed to report a case of hip dysplasia in a rhythmic gymnast surgically managed with arthroscopic retrograde bone grafting, labral repair, cam osteoplasty, double shoelace capsular closure and endoscopic shelf acetabuloplasty. A 20-year-old female college rhythmic gymnast presented complaining of right hip pain and discomfort for the past 2 months. This case report describes the use of the CROSSTRAC guide system to perform retrograde bone grafting to treat the bone cyst at the femoral head arthroscopically. Hip arthroscopic retrograde bone grafting, labral repair, cam osteoplasty, double shoelace capsular closure and endoscopic shelf acetabuloplasty are less invasive and beneficial for the treatment of bone cysts of the femoral head associated with hip dysplasia in symptomatic rhythmic gymnasts.

## INTRODUCTION

Hip dysplasia has been recognized as a cause of labral tears, resulting in hip instability and cartilage damage, predisposing patients to osteoarthritis [1]. Bone cysts in the femoral head are sometimes observed in patients with acetabular dysplasia [2]. There have been several reports demonstrating surgical procedures for treating bone cysts in the femoral head. Jamali *et al*. [3] discussed a procedure using a metal arthroscopic cannula for bone grafting. Günther *et al*. [4] reported the use of an arthroscopically assisted mini-open approach for treating large bone cysts at the femoral head neck junction in three patients in the setting of femoroacetabular impingement (FAI). They reported the common open procedure of antegrade bone grafting.

Shelf acetabuloplasty has been well designed to increase acetabular coverage of the femoral head via the reconstruction of corticocancellous bone grafts on the anterosuperior aspect of the acetabulum. Although favorable clinical outcomes have been reported following open or endoscopic shelf acetabular procedures, periacetabular osteotomy has been the preferred treatment for many surgeons. Recent studies have reported significant improvements in patient-reported outcomes after endoscopic shelf acetabuloplasty for treating patients with acetabular dysplasia combined with rim stress fracture and osteochondritis dissecans [5, 6].

However, recent literature lacks a clear understanding of how to manage bone cysts associated with hip dysplasia. This article aimed to report a case of hip dysplasia in a rhythmic gymnast surgically managed with arthroscopic retrograde bone grafting, labral repair, cam osteoplasty, double shoelace capsular closure and endoscopic shelf acetabuloplasty.

## CASE REPORT

A 20-year-old female college rhythmic gymnast presented complaining of right hip pain and discomfort for the past 2 months. She had no acute trauma and no history of rheumatic disease. She has been playing rhythmic gymnastics for 14 years. The pain was located in the anterior and medial aspect of the right groin. The affected hip of the patient had a limited range of motion (ROM), with 90° of hip flexion and 40° of abduction. With the hip in 90° of flexion in the supine position, the patient had 5° of external rotation and 10° of internal rotation. Pain was reproduced upon performing the flexion adduction and internal rotation (FADIR) test. A hip dial test was performed with the patient in the supine position, and the affected lower limb was manually rotated internally and released for external rotation. External rotation was found to be greater than 45° by the examiner, a positive finding.

**Fig. 1.**
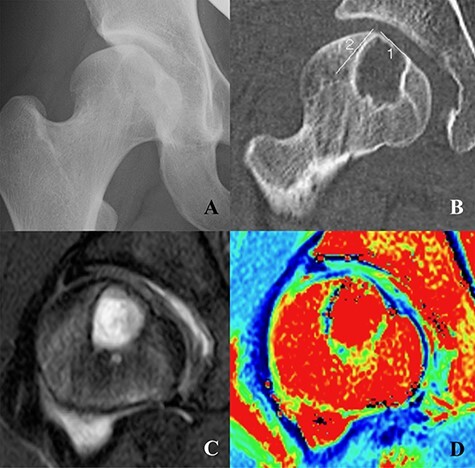
**(A)** A round osteolytic lesion suggesting a large bone cyst at the superior portion of the right femoral head. **(B)** Preoperative coronal CT showing a round bone cyst at the femoral head. **(C)** Coronal MRI showing a high-intensity round lesion at the femoral head. **(D)** T2 mapping showing cartilage of the femoral head. There was no evidence of cartilage damage.

Anteroposterior (AP) pelvic and Dunn radiological views showed a lateral center-edge (LCE) angle of 18°, a Sharp angle of 46°, a Tönnis angle of 23°, an alpha angle of 69°, an offset ratio of 0.09 and absence of the crossover sign (Fig. 1A). Computed tomography (CT) showed a large osteolytic lesion (size: 14 × 20 mm) in the femoral head, suggesting a bone cyst (Fig. 1B). Magnetic resonance imaging (MRI) confirmed a bone cyst at the femoral head, an anterosuperior labral tear and the presence of a cam lesion. There appeared to be no evidence of cartilage damage at the joint surface adjacent to the bone cyst (Fig. 1C and D).

### Surgical technique

**Fig. 2.**
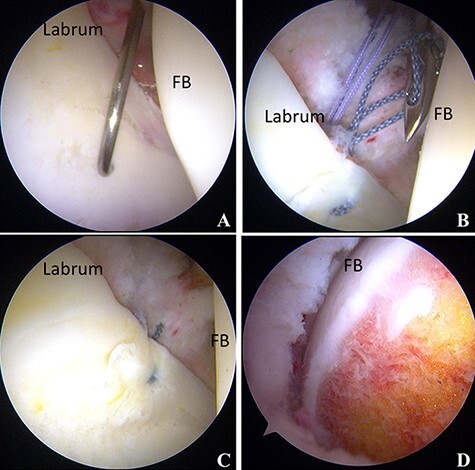
**(A)** Arthroscopic finding from the anterolateral portal showing an anterosuperior labral tear. **(B)** Labral repair was performed with suture anchor fixation. **(C)** Repaired labrum. **(D)** Cam osteoplasty was performed. FH: femoral head.

**Fig. 3.**
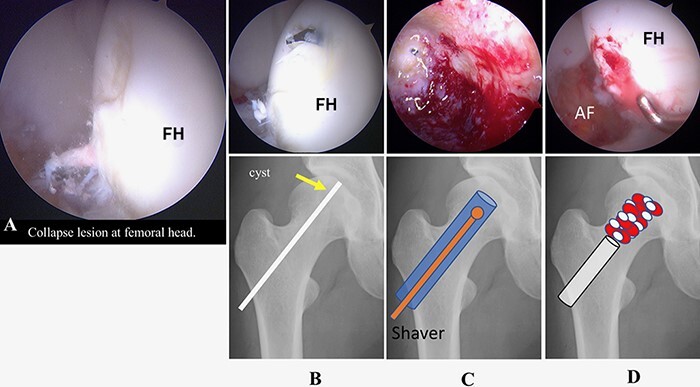
**(A)** Arthroscopic findings showing partial slight collapse of the articular surface of the femoral head. **(B)** A 2.4-mm K-wire was inserted from the greater trochanter to the cystic lesion. **(C)** Fenestration was performed with a Φ10-mm Trecore drill system. Then, the bone tunnel was extended to the cystic lesion, which was curetted away using a shaver. **(D)** Bone chips harvested from the ipsilateral iliac crest and a large amount of β-TCP artificial bone were mixed and inserted into the cavity. FH: femoral head.

**Fig. 4.**
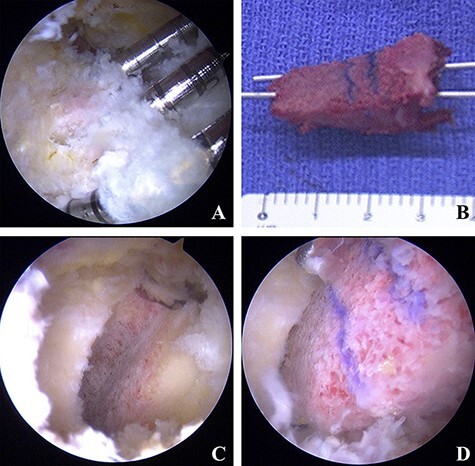
Endoscopic shelf acetabuloplasty. **(A)** Three guide pins (2.4 mm) were inserted along the anterior capsule. **(B)** Free body grafts were harvested from the ipsilateral iliac crest, and 2 parallel 1.5-mm Kirschner wires were inserted. **(C)** An optimally sized shelf slot was made. **(D)** The autologous bone graft was inserted into the slot through the guide wires with press-fit fixation.

Supine hip arthroscopic surgery was performed on a traction table (Hip Positioning System, Smith & Nephew Endoscopy, Andover, MA) with a well-padded peroneal post under general anesthesia. An anterolateral portal (ALP), mid-anterior portal (MAP) and proximal mid-anterior portal (PMAP) were created. A 70°—arthroscope was introduced through the ALP. Interportal capsular release was performed using a beaver knife and radiofrequency (RF) probe for improved scope and instrument accessibility. Intra-articular pathologies were assessed. A labral tear was found from 12:00 to 2:30, and cartilage delamination at the rim lesion was found and classified as multicenter arthroscopy hip outcome research network (MAHORN) grade 2 (Fig. 2A). Labral repair with a suture anchor was performed (Fig. 2B and C). After releasing traction, a dynamic assessment was performed to confirm cam impingement. Then, cam osteoplasty was performed (Fig. 2D). After applying traction again, a collapse lesion at the femoral head was observed in the weight-bearing area and the perifoveal area of the femoral head. Next, the condition of the cartilage was evaluated using a probe, and the cartilage near the bone cyst was found to be collapsed at the anterosuperior portion of the femoral head. Under fluoroscopic guidance, the CROSSTRAC guide system (Smith & Nephew Andover, MA) was placed at the center of the bone cyst and a 2.4-mm guide wire was drilled from the greater trochanter under direct visualization from the MAP. Bone graft material was harvested from the ipsilateral iliac crest. For the retrograde bone grafting procedure, a core reamer was utilized to make a graft tunnel through the 2.4-mm guide wire from the greater trochanter to reach the articular surface of the central portion of the femoral head (Fig. 3A). The autologous cancellous bone tips were delivered through the tunnel by using a bone tunnel dilator from the greater trochanter, and then cylindrical β-TCP (SUPER PORE EX; HOYA Technosurgical, Tokyo, Japan) was placed in the tunnel (Fig. 3). Shoelace capsular plication with Ultratape was performed. After capsular plication, a scope was introduced outside of the capsule. Endoscopic shelf acetabuloplasty was performed [7] (Fig. 4).

**Fig. 5.**
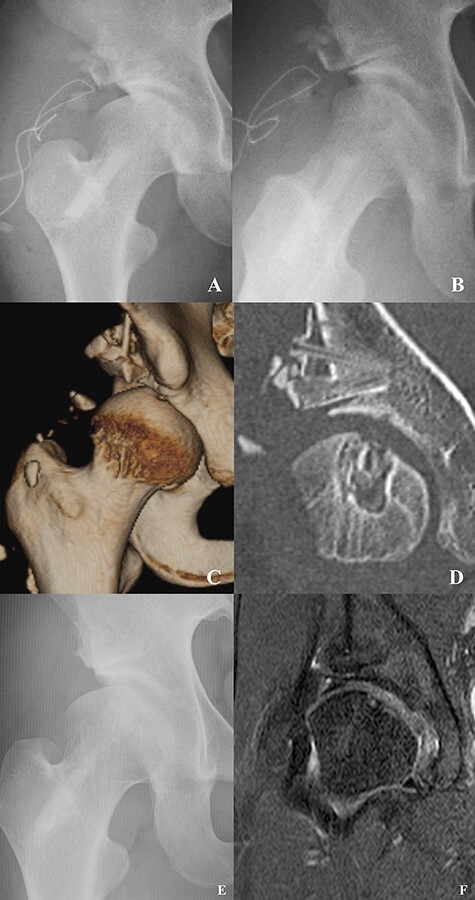
Postoperative **(A)** anteroposterior (AP) pelvic and **(B)** Dunn views showing improved lateral coverage of the acetabulum and femoral head neck offset. **(C)** Postoperative three-dimensional CT showing improved anterosuperior acetabular coverage. **(D)** Postoperative coronal CT showing bone graft incorporation and filling of the bone cyst. **(E)** AP pelvic view at 2 years after surgery showing the shelf graft was remodeling and no evidence of bone cyst in the femoral head. **(F)** The proton density fat suppression coronal view showing complete healing of the femoral head and the shelf graft was adapted.

Postoperative X-ray showed good healing of the bone cyst and appropriate positioning of the shelf graft (Fig. 5A and B).

### Postoperative recovery

Initial non-weight-bearing ambulation using dual crutches for 4 weeks was advanced to partial weight bearing for another 2 weeks. Active hip flexion was limited for 6 weeks to reduce the incidence of hip flexor tendinitis. At 6 weeks postoperatively, full weight bearing was permitted. Postoperative three-dimensional CT showed improved anterosuperior acetabular coverage (Fig. 5C), and the coronal view showed bone graft incorporation and filling of the bone cyst (Fig. 5D). At 4 months after surgery, the patient started jogging, and she returned to rhythmic gymnastics at 9 months after surgery. At 2 years after surgery, X-ray and MRI examinations showed complete healing of the bone cyst in the femoral head where the bone graft was placed (Fig. 5E and F). Physical examination at the time of the last observation confirmed that the hip range of motion was as follows: flexion, 120°; abduction, 60°; adduction, 10°; internal rotation, 60°; and external rotation, 60°. There was no apparent difference between the healthy and affected sides.

## DISCUSSION

In this report, we demonstrated a case of a large bone cyst at the femoral head in the setting of hip dysplasia treated with arthroscopic retrograde bone grafting, labral repair, cam osteoplasty, double shoelace capsular closure and shelf acetabuloplasty.

A number of reports have described subchondral cysts in hip joints. Previous studies have posited that subchondral cysts are formed by the hydrodynamic theory, related to synovial fluid and the mechanical overload theory, related to osseous contusion. Leunig *et al*. [7] hypothesized that cysts form under direct force between the acetabulum and femoral head. He reported that cysts at the femoral head were identified in 33% of FAI cases, and none were identified in dysplastic hips [7]. On the other hand, Inui *et al*. [8] found that 55 of 150 hips had subchondral cysts at the femoral head in dysplastic hips with a narrowing joint space. In the present case, the patient had not been diagnosed with FAI and had no osteoarthritis. From the aforementioned evidence, we considered instability of the femoral head resulting from hip dysplasia and labral tears and hypermobility in the rhythmic gymnast as causes of the bone cyst at the femoral head in this case.

If the bone cyst is not located on a weight-bearing surface, a direct approach to the cystic area can be used for healing. Sharfman *et al*. [9] used a curette and shaver for direct curettage of the bone cyst and then filled the cavity with bone substitute.

In this case, the large cyst was located at the weight-bearing surface of the femoral head. Preoperative MRI showed that the cartilage surface was still preserved. A recent technical note reported a surgical technique for arthroscopic retrograde autograft transplantation for osteochondritis dissecans with a bone cyst at the femoral head [10].

In that case, the cartilage of the femoral head was detached and autologous osteochondral grafting was performed. Similarly, in our case, the remaining cartilage was confirmed arthroscopically, so the large bone cyst at the femoral head could be treated with bone grafting. To protect the remaining cartilage of the femoral head, bone grafting was performed using a retrograde approach to the large bone cyst at the femoral head. Morattel *et al*. [11] used a similar approach to treat apical femoral head deformities. We consider that this surgical technique can be useful for the treatment of other types of diseases.

In this case, endoscopic shelf acetabuloplasty was performed for the dysplasia that caused the bone cyst. It has been reported that damage to the ligaments is involved in the formation of bone cysts [12]. However, we believe that the shelf was necessary in this case. Anteroposterior (AP) pelvic and Dunn radiological views showed a LCE angle of 18°, a Sharp angle of 46° and a Tonnis angle of 23°. This patient was categorized as having borderline hip dysplasia. Several studies have reported favorable clinical outcomes after hip arthroscopic labral preservation. However, Hatakeyama *et al*. [13] demonstrated that preoperative predictors of poorer outcomes after hip arthroscopic labral preservation, capsular plication and cam osteoplasty in the setting of borderline developmental dysplasia of the hip include age ≥42 years old, a broken Shenton line, osteoarthritis, a Tonnis angle greater than 15° and a VCA angle less than 17° on preoperative radiographs.

Since this patient had a Tonnis angle of 23°, we do not consider this patient to have benefit from isolated hip arthroscopic labral preservation and capsular plication. Thus, endoscopic shelf acetabuloplasty is indicated for this patient.

Uchida *et al*. [14] reported that endoscopic shelf acetabuloplasty concomitant with labral repair, cam osteoplasty and capsular plication can yield good clinical outcomes, with a high rate of return to sports for artistic dancers in the setting of acetabular dysplasia. This case report lends support to this evidence.

The indication for the retrograde bone grafting technique is a bone cyst at the femoral head. A contraindication of this technique is an infection of the surrounding hip joint and osteoarthritis (Table I).

**Table I.** Indications for and contraindications to retrograde bone grafting

*Indications*	*Contraindications*
Bone cyst at the femoral head Cartilage is healthy and not detached	Infection surrounding hip joint
	Osteoarthritis

Arthroscopic bone grafting to treat bone cysts at the femoral head has several advantages and some disadvantages. The advantages are that this procedure is minimally invasive and has the potential to allow early rehabilitation and quicker recovery. Concurrent lesions can be readily assessed and treated arthroscopically. Surgeons are able to assess impingement issues and other intra-articular pathologies, such as acetabular labral tears, ligament tears and cartilage delamination, since developmental dysplasia of the hip (DDH) is often associated with other pathologies. However, this procedure is meticulous and technically demanding, which are considerable disadvantages (Table II).

**Table II.** Advantages and disadvantages of retrograde bone grafting

*Advantages*	*Disadvantages*
Minimally invasive	Technically demanding
Few severe complications	Possibility of all complications of hip arthroscopic surgery Potential risk of iatrogenic cartilage damage
Ability to assess impingement issues and other intra-articular pathologies	
Early rehabilitation	
Quick recovery	

Surgical pearls and pitfalls for this technique are detailed in Table III.

**Table III.** Pearls and pitfalls

*Pearls*	*Pitfalls*
Preoperative evaluation and planning with computed tomography is indispensable	Treating massive collapsed lesions and damage at the articular surface is difficult
The position of the collapsed lesion at the femoral head can be identified under arthroscopy	The CROSSTRAC system can only be used if the bone cyst is located in the joint
The CROSSTRAC system is placed at the center of the bone cyst lesion, and a 2.4-mm guide wire is drilled from the greater trochanter under direct visualization from the MAP under fluoroscopy	
A core reamer is utilized to make a graft tunnel from the greater trochanter to the bone cyst in femoral head	
The autologous cancerous bone tips are delivered through the tunnel by using a bone tunnel dilator from the greater trochanter, and then cylindrical β-TCP is placed into the tunnel	

## CONCLUSION

Hip arthroscopic retrograde bone grafting, labral repair, cam osteoplasty, double shoelace capsular closure and endoscopic shelf acetabuloplasty are less invasive and beneficial for the treatment of bone cysts at the femoral head associated with hip dysplasia in symptomatic rhythmic gymnasts.

## DATA AVAIILABILITY

The patient provided informed consent for the publication of related to this case.
